# Omadacycline is active *in vitro* and *in vivo* against ciprofloxacin-resistant *Bacillus anthracis*

**DOI:** 10.1128/aac.00595-24

**Published:** 2024-08-12

**Authors:** Henry S. Heine, George Drusano, Bret K. Purcell, Diane Anastasiou, S. Ken Tanaka, Alisa W. Serio

**Affiliations:** 1 University of Florida, Orlando, Florida, USA; 2 Paratek Pharmaceuticals, Inc., King of Prussia, Pennsylvania, USA; The Peter Doherty Institute for Infection and Immunity, Melbourne, Australia

**Keywords:** omadacycline, *Bacillus anthracis*, inhalation anthrax

## Abstract

*Bacillus anthracis*, the causative agent of anthrax, is among the most likely bacterial pathogens to be used in a biological attack. Inhalation anthrax is a serious, life-threatening form of infection, and the mortality from acute inhaled anthrax can approach 100% if not treated early and aggressively. Food and Drug Administration-approved antibiotics indicated for post-exposure prophylaxis (PEP) or treatment of anthrax are limited. This study assessed the *in vitro* activity and *in vivo* efficacy of omadacycline and comparators against clinical isolates of *B. anthracis,* including a ciprofloxacin-resistant isolate. Minimum inhibitory concentrations (MICs) of omadacycline, ciprofloxacin, and doxycycline were determined against animal and human clinical isolates of *B. anthracis*, including the ciprofloxacin-resistant Ames strain BAC^r^4-2. Mice were challenged with aerosolized BAC^r^4-2 spores, and survival was monitored for 28 days post-challenge. Treatment was initiated 24 h after aerosol challenge and administered for 14 days. Omadacycline demonstrated *in vitro* activity against 53 *B. anthracis* isolates with an MIC range of ≤0.008–0.25 µg/mL, and an MIC_50_/MIC_90_ of 0.015/0.03 µg/mL. Consistent with this, omadacycline demonstrated *in vivo* efficacy in a PEP mouse model of inhalation anthrax caused by the Ames BAC^r^4-2 ciprofloxacin-resistant *B. anthracis* isolate. Omadacycline treatment significantly increased survival compared with the vehicle control group and the ciprofloxacin treatment group. As antibiotic resistance rates continue to rise worldwide, omadacycline may offer an alternative PEP or treatment option against inhalation anthrax, including anthrax caused by antibiotic-resistant *B. anthracis*.

## INTRODUCTION

*Bacillus anthracis* is the causative agent of anthrax and is classified as a Category A biothreat pathogen (the highest risk), Tier 1 select agent (1, 2). This organism has the potential to be mass produced and mass dispersed via aerosolization of spores. Consequently, it is considered to be one of the most likely agents to be weaponized for use in a biological attack (1), thus posing a significant risk to public health and security (1). Anthrax is a naturally occurring zoonotic disease, with cutaneous anthrax being the most common form observed in humans, whereas systemic anthrax resulting from inhalation of spores accounts for <5% of cases (2). However, inhalation anthrax is the most serious, life-threatening form of the disease (3) and is the most likely route of biological attack. If left untreated, mortality from acute inhaled anthrax can approach 100% (4).

Food and Drug Administration (FDA)-approved antibiotics indicated for post-exposure prophylaxis (PEP) or treatment of anthrax are limited (1). The Centers for Disease Control and Prevention (CDC) guidelines for the prevention and treatment of anthrax list first-line and alternative antimicrobial drug treatment regimens for non-pregnant adults and other patient groups for several types of anthrax, including systemic and cutaneous (1). First-line agents for PEP for non-pregnant adults include doxycycline, fluoroquinolones (ciprofloxacin or levofloxacin), and penicillin-class agents, amoxicillin and penicillin (1). Empiric treatment recommendations for non-pregnant adults list these same antimicrobials with the addition of minocycline and carbapenems (meropenem, imipenem), and highlight the need for combination antimicrobial therapy using drugs with different mechanisms of action (1). Although the penicillin-class agents are listed, the causative pathogen must be penicillin-susceptible (1). Further, the use of fluoroquinolones is limited due to safety concerns, which has prompted a black box warning from the FDA (1, 5–7). In addition, the threat of antibiotic resistance, whether naturally occurring or via genetic engineering, threatens the efficacy of first-line agents (8, 9).

Therefore, further studies and the future availability of new first-line antimicrobial agents are critical to bolster the current armamentarium, particularly against resistant strains, which would prove useful in the event of an accidental or intentional release of resistant pathogens (10). Omadacycline is a semisynthetic tetracycline-class antibiotic that was designed to overcome the two most common tetracycline class resistance mechanisms: ribosomal protection proteins and tetracycline-specific efflux pumps (11, 12). Similar to other tetracyclines, omadacycline inhibits bacterial protein synthesis by binding to the 30S ribosomal subunit. Omadacycline is approved by the US FDA for the treatment of adults with acute bacterial skin and skin structure infections (ABSSSI) and community-acquired bacterial pneumonia (CABP) and is available as a once-daily intravenous (IV) or bioequivalent oral formulation (13). Omadacycline is being developed under US FDA Animal Rule guidance (14) for PEP and treatment of inhalation anthrax based on previously demonstrated potent *in vitro* activity against *B. anthracis*, as well as *in vivo* efficacy in mouse (PEP and treatment) and rabbit (treatment) models of inhalation anthrax (10, 15–17). Of note, omadacycline is listed in the 2023 CDC guidelines as a potentially effective antimicrobial drug for PEP and treatment of anthrax, presumably based on its *in vitro* and *in vivo* activity and its ability to overcome tetracycline class (doxycycline and minocycline) resistance (1).

In this paper, we assessed the ability of omadacycline to overcome *B. anthracis* ciprofloxacin resistance *in vitro* and *in vivo*. Omadacycline *in vitro* activity was assessed against a diverse set of *B. anthracis* animal and human clinical isolates, including a ciprofloxacin-resistant isolate. In addition, the efficacy of omadacycline for PEP against inhalation anthrax caused by a ciprofloxacin-resistant *B. anthracis* isolate was determined in mice. The efficacy of omadacycline was measured by survival and compared to negative (untreated) controls.

## MATERIALS AND METHODS

### Study

#### Bacterial strains and determination of MICs

*B. anthracis* animal and human clinical isolates from America, Africa, Europe, and Russia, including the Ames, Sterne, and Vollum strains and the ciprofloxacin-resistant Ames strain BAC^r^4-2 (18), which is resistant to ciprofloxacin due to a *gyrA* mutation, were evaluated *in vitro* (17). Quality control organisms *Escherichia coli* American Type Culture Collection (ATCC) strain 25922, *Pseudomonas aeruginosa* ATCC 27853, and *Staphylococcus aureus* ATCC 29213 were also evaluated.

Minimum inhibitory concentrations (MICs) were determined via broth microdilution in cation-adjusted Mueller-Hinton broth (CAMHB) according to the Clinical Laboratory Standard Institute (CLSI) methodology (19). Bacterial inocula were prepared by suspension of colonies into CAMHB from 18-h *B. anthracis* sheep blood agar (SBA) plates that were incubated at 35°C. Suspended cultures were each diluted with CAMHB to a bacterial cell density of 10^6^ CFU/mL adjusted based on optical density at 600 nm (OD600). The conversion factor, *B. anthracis* 3.82 × 10^7^ CFU/mL/OD600, was used. To each well of the 96-well plates, 50 µL of the adjusted dilution was added for a final inoculum of approximately 5 × 10^4^ CFU/well in 100 µL. Omadacycline (Paratek Pharmaceuticals, Inc) and comparators (doxycycline, tetracycline, ciprofloxacin, levofloxacin, and moxifloxacin; purchased from US Pharmacopeia) were serially diluted, with concentrations ranging from 16 to 0.008 µg/mL, based on a final well volume of 100 µL after inoculation. MIC values were visually determined after 18-h incubation at 35°C. The MIC values of omadacycline, ciprofloxacin, and doxycycline were also determined with vegetative bacteria germinated from the Ames strain BAC^r^4-2 spore stock before infection of mice.

### Mouse study

#### Mice, ethics, and study conduct

Female BALB/c mice (6–8 weeks) were obtained from Charles River Laboratories, Kingston, NY. All experimental procedures adhered to the guidelines stated in the Guide for the Care and Use of Laboratory Animals (20). Studies were conducted in the BSL-3 laboratories of the University of Florida, Orlando, and complied with the Animal Welfare Act and other federal statutes and regulations relating to animals and experiments involving animals. The experiments were performed under the University of Florida Institutional Animal Care and Use Committee (IACUC) protocol 202006585 and Institutional Biosafety Committee (IBC) approved protocol BA3896. Additionally, the retention and use of this strain were extensively reviewed and approved by the CDC/DSAT (Division of Select Agents and Toxins), Protocol 2431SHAMOUX, before the original 2017 study which was supported by CDC.

#### Preparation of the *B. anthracis BAC^r^4-2* challenge strain for aerosolization

The *B. anthracis* Ames strain BAC^r^4-2 spores were harvested from ≥48-h broth cultures and further purified by sucrose density gradient centrifugation and Ficoll density gradient centrifugation. The spores were stored and maintained at 4°C in sterile water for injection.

#### Aerosol infection

The spores were heat shocked at 65°C for 15 min, and the concentration was adjusted to approximately 1 × 10^10^ CFU/mL with sterile water for injection. Aerosol was generated using a three-jet Collison nebulizer (21), and all aerosol procedures were controlled and monitored using the Automated Bioaerosol Exposure system operating with a whole-body rodent exposure chamber (22). Integrated air samples were obtained from the chamber during each exposure using an all-glass impinger (AGI). Aerosol bacterial samples were serially diluted and plated on SBA. The inhaled dose (CFU/mouse) of *B. anthracis* spores was estimated using Guyton’s formula (23) and calculated according to Roy et al. (24).

Mice were challenged with aerosolized BAC^r^4-2 spores, and survival was monitored for 28 days post-challenge. Treatment was initiated 24 h after aerosol challenge and administered for 14 days. Treatment groups included omadacycline (intraperitoneal [IP] administration, 0.75, 2.5, 3.75, 5, 7.5, and 15 mg/kg every 12 h), ciprofloxacin (IP; 30 mg/kg every 12 h), doxycycline (IP; 2 and 40 mg/kg every 12 h), and vehicle control. All treatment groups (*n* = 10 in each) had five mice from each group included in every aerosol run to proactively minimize the potential effect of spray run variations on study outcomes.

#### Assessment of efficacy

The cohort size for statistical evaluation was 10 mice. Mortality was assessed and recorded 3–4 times daily during antibiotic administration (14 days) and at least twice daily thereafter up to 28 days post-challenge. A grading/evaluation system that scored appearance, natural behavior, and provoked behavior was used to determine euthanasia criteria: 0–2: normal; 3–8: monitor frequently; 9: moribund, euthanize.

#### 28-day blood and tissue bacterial load

All surviving animals from each group were euthanized via CO_2_ exposure. Blood was immediately collected and plated on SBA for CFU evaluation. The animals were necropsied for lung and spleen tissues, which were weighed and homogenized. Lung and spleen homogenates were serially diluted in saline and spread onto SBA. All SBA plates were incubated at 35°C for 18–24 h to determine any remaining bacterial load. The limits of detection were approximately 5 CFU/mL in blood, 17 CFU/g for lung, and 50 CFU/g for spleen.

#### Data analysis

All analyses were performed employing a stratified Kaplan–Meier analysis with a log-rank test as implemented on GraphPad Prism Software, version 6.01. Sample size was based on the minimum sample size required for statistical significance using log-rank analysis of survival (Mantel–Cox) and paired ANOVA (Bonferroni adjustment) of results comparing each experimental arm and vehicle control. The study sample size was validated in previous reports of the use of the murine experimental model for *in vivo* analysis of the efficacy of antimicrobial agents for inhalation plague, tularemia, anthrax, and melioidosis (25–27).

## RESULTS

### *In vitro* study

**Fig 1:**
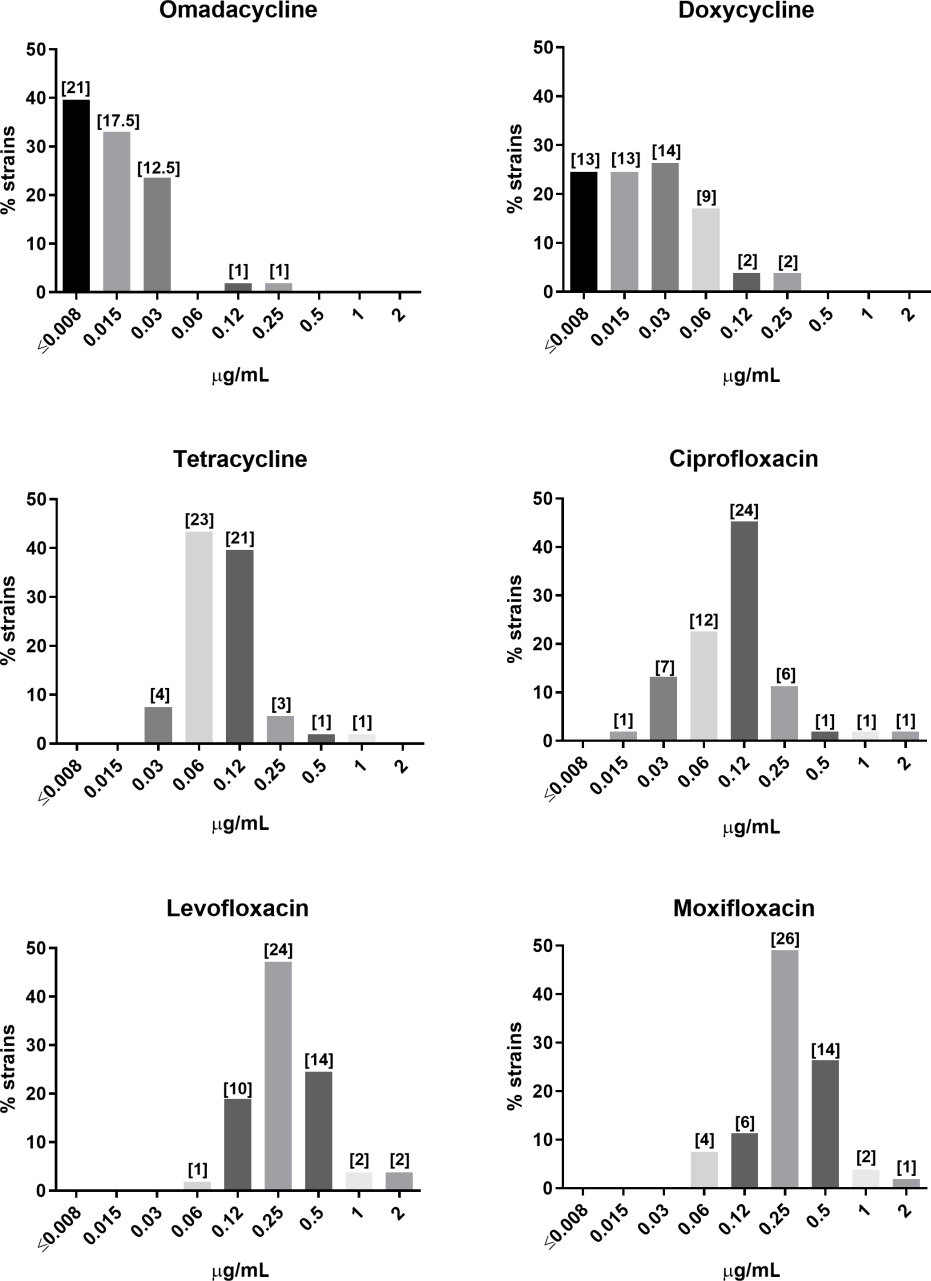
MIC distribution of omadacycline (duplicate testing) and comparator agents against 53 *B. anthracis* isolates. The number of strains per MIC value is shown in brackets. Omadacycline MIC values are an average of duplicate testing.

Omadacycline demonstrated *in vitro* activity against the animal and human clinical *B. anthracis* isolates (*N* = 53) with an MIC range of ≤0.008–0.25 µg/mL, an MIC_50_ of 0.015 µg/mL, and an MIC_90_ of 0.03 µg/mL (Fig. 1; Table 1). Doxycycline and tetracycline also demonstrated activity with MIC_90_ values of 0.06 and 0.25 µg/mL, respectively. Omadacycline MIC values were generally equal to or lower than the doxycycline and tetracycline MIC values. The fluoroquinolones tested (ciprofloxacin, levofloxacin, and moxifloxacin) had MIC_90_ values ranging from 0.25 to 0.5 µg/mL.

**TABLE 1:** *In vitro* minimum inhibitory concentrations of omadacycline and comparators against *B. anthracis* isolates, *N* = 53^*a*^

MIC values, µg/mL	Omadacycline	Doxycycline	Tetracycline	Ciprofloxacin	Levofloxacin	Moxifloxacin
MIC_50_	0.015	0.03	0.12	0.12	0.25	0.25
MIC_90_	0.03	0.06	0.25	0.25	0.5	0.5
Range	≤0.008–0.25	≤0.008–0.25	0.03–1	0.015–2	0.06–2	0.06–2

^*a*^ MIC = minimum inhibitory concentration.

The BAC^r^4-2 ciprofloxacin-resistant strain was evaluated in *in vitro* MIC assays on two occasions. In the first assessment, BAC^r^4-2 was one of 53 isolates evaluated (Table 1), and in the second assessment, BAC^r^4-2 vegetative bacteria germinated from spore stock prior to the challenge was evaluated. The resulting MIC values of ciprofloxacin (2 and 4 µg/mL), omadacycline (0.015 and ≤0.008 µg/mL), and doxycycline (0.03 and 0.015 µg/mL) against the BAC^r^4-2 strain, within the 53-isolate panel and as vegetative bacteria germinated from spore stock, respectively, were similar and within twofold dilution.

### Mouse study

#### 28-day survival

**Fig 2:**
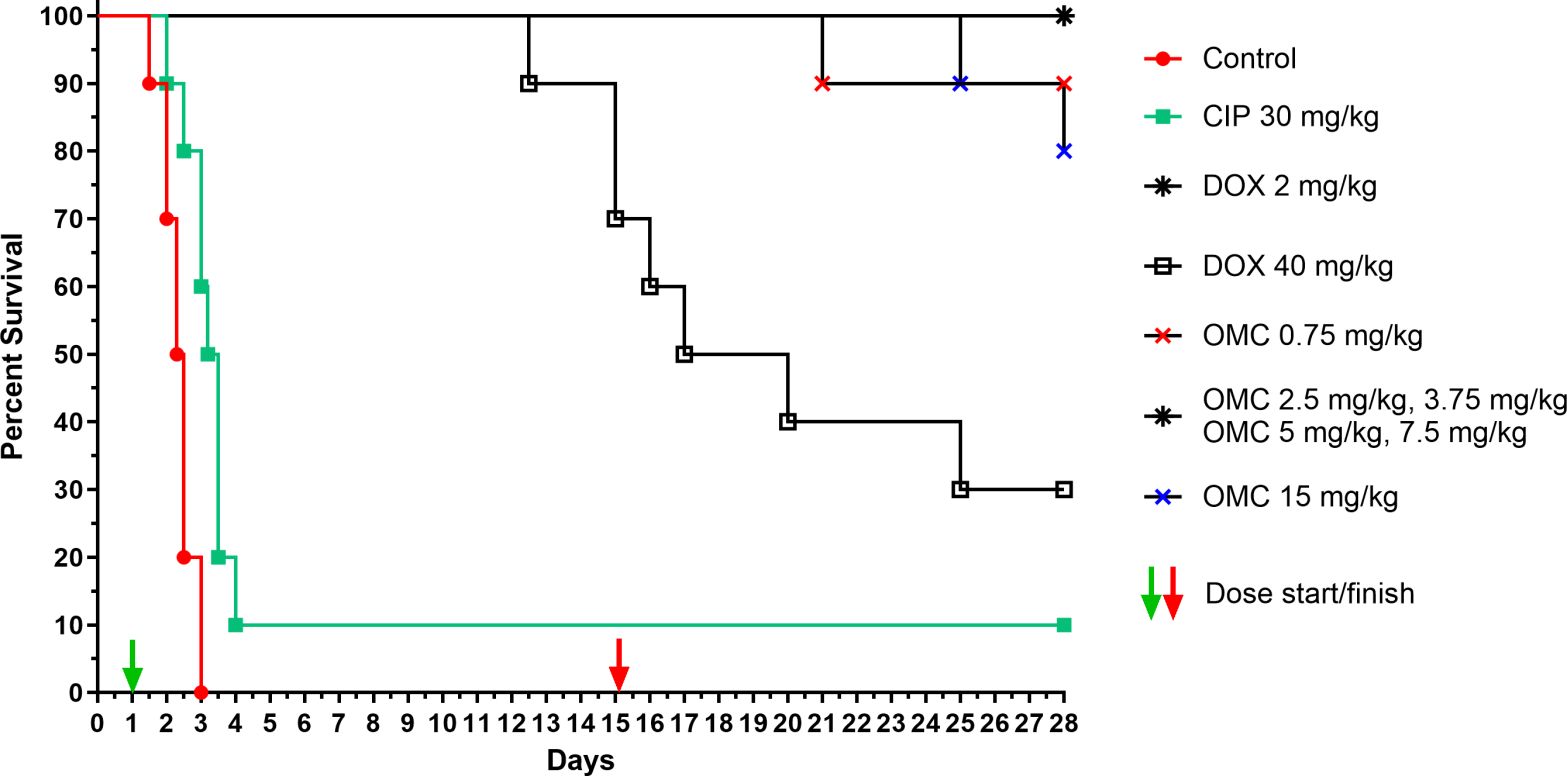
Kaplan–Meier mouse survival data: omadacycline efficacy vs inhalation anthrax caused by ciprofloxacin-resistant *B. anthracis.* All doses were administered q12 h. CIP, ciprofloxacin; DOX, doxycycline; OMC, omadacycline.

Survival curves are presented in Fig. 2. Omadacycline treatment groups of 2.5, 3.75, 5, and 7.5 mg/kg resulted in 100% survival. Omadacycline treatment groups of 0.75 and 15 mg/kg doses had 90% and 80% survival, respectively. The mean time-to-death (MTD) for the omadacycline treatment groups could not be determined as there were ≤2 deaths in any treatment group. Doxycycline 2.5 mg/kg resulted in 100% survival, whereas doxycycline 40 mg/kg resulted in only 30% survival at 28 days, with a MTD of 18.5 days. As expected, ciprofloxacin treatment against anthrax caused by a ciprofloxacin-resistant strain failed, and 90% of mice died, with a MTD of 3.35 days. All animals died in the vehicle control group, with a MTD of 2.4 days. The survival curves for all omadacycline and doxycycline cohorts differed significantly (*P* < 0.0001) from those of the vehicle cohort. The MTD of the ciprofloxacin group was significantly different from the vehicle cohort (*P* = 0.0017), indicating the infection was slowed with treatment, despite resistance.

#### 28-day blood and tissue bacterial load

**Fig 3:**
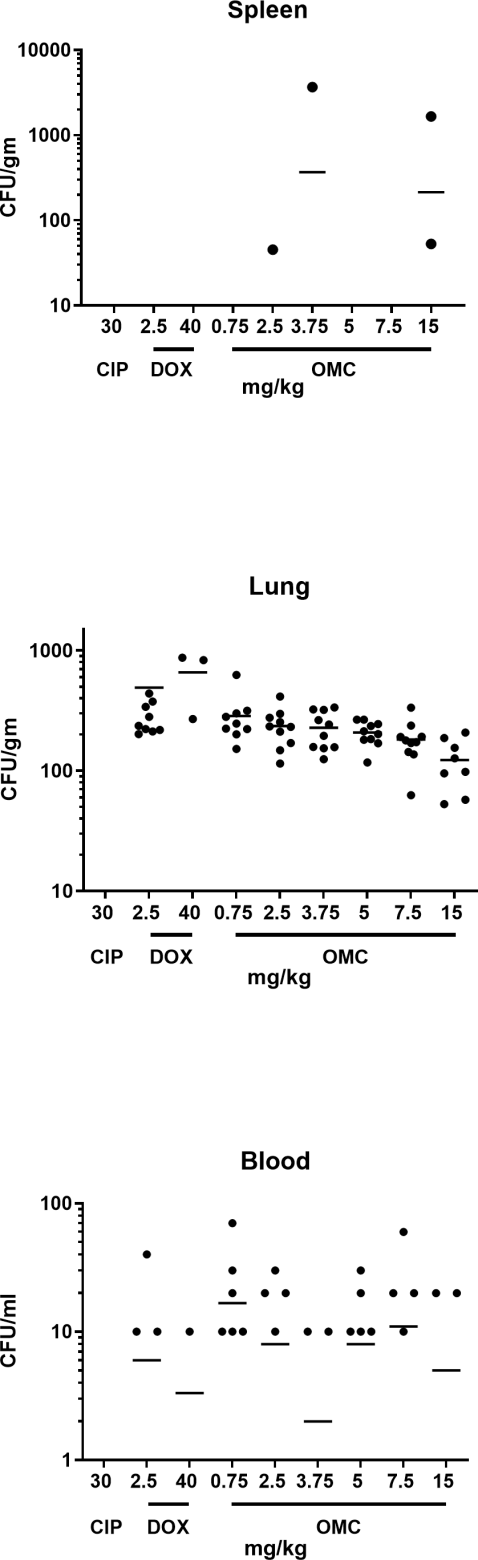
28-day blood and tissue mouse *B. anthracis* bacterial loads. All doses were administered q12 h. Circles represent individual animals that survived to the end of the study, and lines represent the means. CIP, ciprofloxacin; DOX, doxycycline; OMC, omadacycline.

*B. anthracis* bacterial loads in the spleen, lung, and blood of mice surviving at day 28 are presented in Fig. 3. The limits of detection are approximately 5 CFU/mL in blood, 17 CFU/g for the lung, and 50 CFU/g for the spleen. Bacterial load was below the limit of detection for most of the blood and spleens from mice surviving at 28 days. Lung tissue loads were all below the 10^5^ CFU/g tissue previously observed to be the limit of reinfection (25). No gross pathology was observed during tissue harvest except for the 40 mg/kg doxycycline animals, where the peritonea had large, granulomatous-like lesions.

## DISCUSSION

Omadacycline demonstrated potent *in vitro* activity against a diverse collection of *B. anthracis* isolates, consistent with previous reports (10, 17). The omadacycline MIC_90_ value was twofold lower than the doxycycline MIC_90_ value (0.03 µg/mL vs 0.06 µg/mL, respectively). However, all of the omadacycline and doxycycline MIC values ranged from ≤0.008 to 0.25 µg/mL, where the tetracycline MIC values ranged fourfold higher (0.03 to 1 µg/mL). Ciprofloxacin, levofloxacin, and moxifloxacin had similar activity to each other, with MIC_90_ values ranging from 0.25 to 0.5 µg/mL, with ciprofloxacin slightly more potent than the other two drugs, with a broader MIC range (i.e., 0.015 to 2 µg/mL vs 0.06 to 2 µg/mL, respectively). As previously demonstrated, the Ames strain BAC^r^4-2 was confirmed to be ciprofloxacin resistant *in vitro* with MIC values of 2 to 4 µg/mL (15, 18). Although the MIC values reported in this study are slightly lower than those previously reported [MIC of 16 µg/mL (18)], these results confirm resistance to ciprofloxacin based on CLSI MIC interpretive criteria, i.e., ciprofloxacin susceptible at MIC values ≤0.25 µg/mL (28). Omadacycline remained active against this isolate, with replicate MIC values of ≤0.008 and 0.015 µg/mL, similar to that observed for the ciprofloxacin-susceptible Ames strain (≤0.015 µg/mL) (15), confirming that ciprofloxacin resistance mediated by a *gyrA* mutation does not impact omadacycline activity. Of note, all of the comparator MIC values were within ranges previously observed against these strains (10, 17).

Consistent with the *in vitro* data, omadacycline demonstrated *in vivo* efficacy in a PEP mouse model of inhalation anthrax caused by the Ames BAC^r^4-2 ciprofloxacin-resistant *B. anthracis* isolates. Omadacycline treatment significantly increased survival compared with the vehicle control group and ciprofloxacin treatment group. The results from this study are consistent with results from a previous study (10) that demonstrated the efficacy of omadacycline at all doses tested in a mouse model of inhalation anthrax caused by a ciprofloxacin-susceptible *B. anthracis* strain, confirming that omadacycline maintains the efficacy in this model regardless of ciprofloxacin resistance. In addition, the doxycycline results observed in this study for the 2.5 mg/kg dose were also comparable to those in previous studies (10, 25). However, there were deaths observed in the 40 mg/kg doxycycline treatment group, which appears to be due to the antibiotic dose itself and not a failure of the treatment. Because the cause of death was not due to anthrax, it was beyond the scope of this investigation to investigate further. However, this observation is consistent with another study reporting doxycycline-induced cardiomyopathy in rats receiving high doses of doxycycline (50 mg/kg) twice daily (29). Finally, the failure of ciprofloxacin treatment in this study due to ciprofloxacin resistance was consistent with a previous report (18). The *B. anthracis* bacterial loads observed in the tissues of surviving mice at the end of the study are most likely due to ungerminated spores as observed in past studies (18, 25).

Currently, the FDA-approved antibiotics for PEP of inhalation anthrax include doxycycline, ciprofloxacin, levofloxacin, and penicillin G procaine, with ciprofloxacin and doxycycline recommended as first-line agents. Due to the lack of long-term (>30 days) safety data, levofloxacin was not recommended as first-line (1). Amoxicillin and penicillin VK are also listed as first-line antimicrobials, but only if the *B. anthracis* causative pathogen is penicillin-susceptible (1). There are limited FDA-approved antibiotics for the treatment of inhalation anthrax (i.e., penicillin), but treatment guidelines exist. The CDC guidelines recommend that empiric treatment for systemic disease with or without suspected meningitis relies on ≥3 and ≥2 antimicrobials with different mechanisms of action for treatment. The guidelines also provide recommendations for first-line agents, which often overlap with those recommended for PEP (1). However, there are limitations to these PEP and treatment options, for example, black box warnings exist for the fluoroquinolones (e.g., ciprofloxacin), and there is a risk for the development of resistance on therapy with beta-lactams (e.g., penicillin) (6, 30). These limitations highlight the difficulty that could be faced if mass distribution of the currently listed first-line agents is required. Furthermore, antibiotic resistance rates continue to rise worldwide (31), and the availability of molecular tools could enable genetic engineering of *B. anthracis* strains to be resistant to drugs, such as fluoroquinolones and doxycycline (8, 9). Therefore, there is a need for alternative first-line PEP and treatment options.

Omadacycline was designed to overcome the most common tetracycline class resistance mechanisms and is also not impacted by ciprofloxacin resistance. The 2023 CDC Guidelines for the Prevention and Treatment of Anthrax lists omadacycline as an alternative antimicrobial agent for PEP and treatment of anthrax infections and was highlighted as a “potentially effective option for certain tetracycline-resistance mechanisms (e.g., efflux pumps)” (1), as has been demonstrated for other Gram-positive pathogens *in vitro* and *in vivo* (12). In addition, as a protein synthesis inhibitor, omadacycline might also contribute to a possible reduction of toxins (11, 32, 33).

This study has some limitations. The data from *in vitro* and *in vivo* studies suggest that omadacycline has the potential to be effective in PEP or treatment of inhalation anthrax in humans, but additional data are needed. Although mice have been extensively used in anthrax research, they are considered proof-of-concept models because responses to the infection might differ between mice and humans (34). The doses of omadacycline used in this study were effective in the mouse model and in all omadacycline treatment arms at or below 15 mg/kg q12 h, which was previously shown to be the dose used in BALB/c mice that best represented the human area under the curve (35). Therefore, this study represents a proof of concept, regarding the potential usefulness of omadacycline in inhalation anthrax, particularly when the issue of antibiotic resistance is considered.

### Conclusions

Omadacycline may offer an alternative PEP or treatment option in the event of an anthrax bioterror attack or unintentional exposure. Together, the data from these *in vitro* and *in vivo* studies support further development of omadacycline as a potential PEP and treatment option against inhalation anthrax, including anthrax caused by antibiotic-resistant *B. anthracis*.
